# Unusual Presentation of Non-Gestational Extragonadal Choriocarcinoma

**DOI:** 10.7759/cureus.74072

**Published:** 2024-11-20

**Authors:** Islam Gafar, Mohamed Elhassan, Ammar Elhaj, Paula Calvert

**Affiliations:** 1 General Medicine, University Hospital Waterford, Waterford, IRL; 2 Oncology, University Hospital Waterford, Waterford, IRL

**Keywords:** choriocarcinoma syndrome, extragenital choriocarcinoma, extra-gestational choriocarcinoma, extra gonadal choriocarcinoma, non-gestational choriocarcinoma

## Abstract

Non-gestational choriocarcinoma is an extremely rare and highly aggressive malignant tumor that arises independent of gestational events, making less than 0.6% of all ovarian germ cell tumors. Unlike the more common gestational choriocarcinoma, which is associated with pregnancy, non-gestational choriocarcinoma originates from germ cells within the ovary. It accounts for a small fraction of ovarian malignancies and is often characterized by elevated levels of serum beta-human chorionic gonadotropin (β-HCG). The rarity and clinical overlap with other ovarian tumors pose significant diagnostic challenges, necessitating a thorough histopathological and immunohistochemical examination for accurate diagnosis. A 39-year-old female presented with a two-week history of right-sided migraine and general malaise, followed by a three-day onset of vision loss in the right eye. Initial evaluation in the emergency department included a chest X-ray, which revealed a 10 cm rounded opacity in the upper lobe of the left lung. A CT scan of the head showed a 4.5 cm rim-enhancing lesion in the left occipital lobe, along with a left middle cerebral artery (MCA) aneurysm. Notably, her serum β-HCG levels were significantly elevated at 5,642 mIU/mL despite the absence of intrauterine or extrauterine pregnancy on abdominal and pelvic ultrasound.

Further workup included a CT thorax and MRI of the brain, which confirmed the isolated lung mass and left occipital lobe mass with no other sites of disease, leading to her transfer to the neurosurgery department. The patient underwent a left occipital craniotomy with tumor resection. Histopathological analysis confirmed the diagnosis of choriocarcinoma. Chromosomal analysis showed no evidence of the Y chromosome and confirmed the non-gestational origin of the choriocarcinoma. This case report discusses the non-specific presentation, radiological features, current treatment options, and potential safety strategies for managing this rare condition.

## Introduction

Ovarian choriocarcinoma is an extremely rare and aggressive cancer, making up less than 1% of ovarian germ-cell tumors. It can arise from gestational tissue (gestational choriocarcinoma (GCO)) or from ovarian germ cells (non-gestational choriocarcinoma (NGCO)). GCO is exceptionally rare, occurring approximately one per 100,000 pregnancies, with a higher incidence depending on increasing maternal age, while NGCO accounts for less than 0.6% of ovarian germ-cell tumors. Proper diagnosis is critical, as the treatment approach and prognosis differ significantly between GCO and NGCO [1,2].

GCO is linked to a patient's pregnancy history and can coexist with a well-developed corpus luteum. Cure rates can reach up to 90% with single-agent chemotherapy, typically using methotrexate [3]. On the other hand, NGCO may originate from germ cells in the gonads or extragonadal sites and can occasionally arise in parenchymal organs due to dedifferentiation of somatic carcinoma [4]. Non-gestational and mixed choriocarcinomas are generally more aggressive than their gestational counterparts. These tumors frequently present with metastases and have a poorer clinical outcome, leading to lower survival rates. Consequently, a more intensive chemotherapy regimen is often necessary to address the severity of these cases. Therefore, it is crucial to differentiate between gestational and non-gestational origins. DNA polymorphism analysis is essential in guiding the appropriate chemotherapy regimen [5,6].

## Case presentation

A 39-year-old female presented to the regional hospital with a two-week history of right-sided migraine and general malaise, followed by a four-day history of flashing red light in her right eye, progressing to vision loss in the same eye for three days. Upon further evaluation, her medical and surgical history was unremarkable, with no previous vaginal bleeding, pregnancies, or miscarriages. She is sexually active with one partner, has a seven-pack-year smoking history, and denies intravenous drug use or B symptoms. 

**Figure 1:**
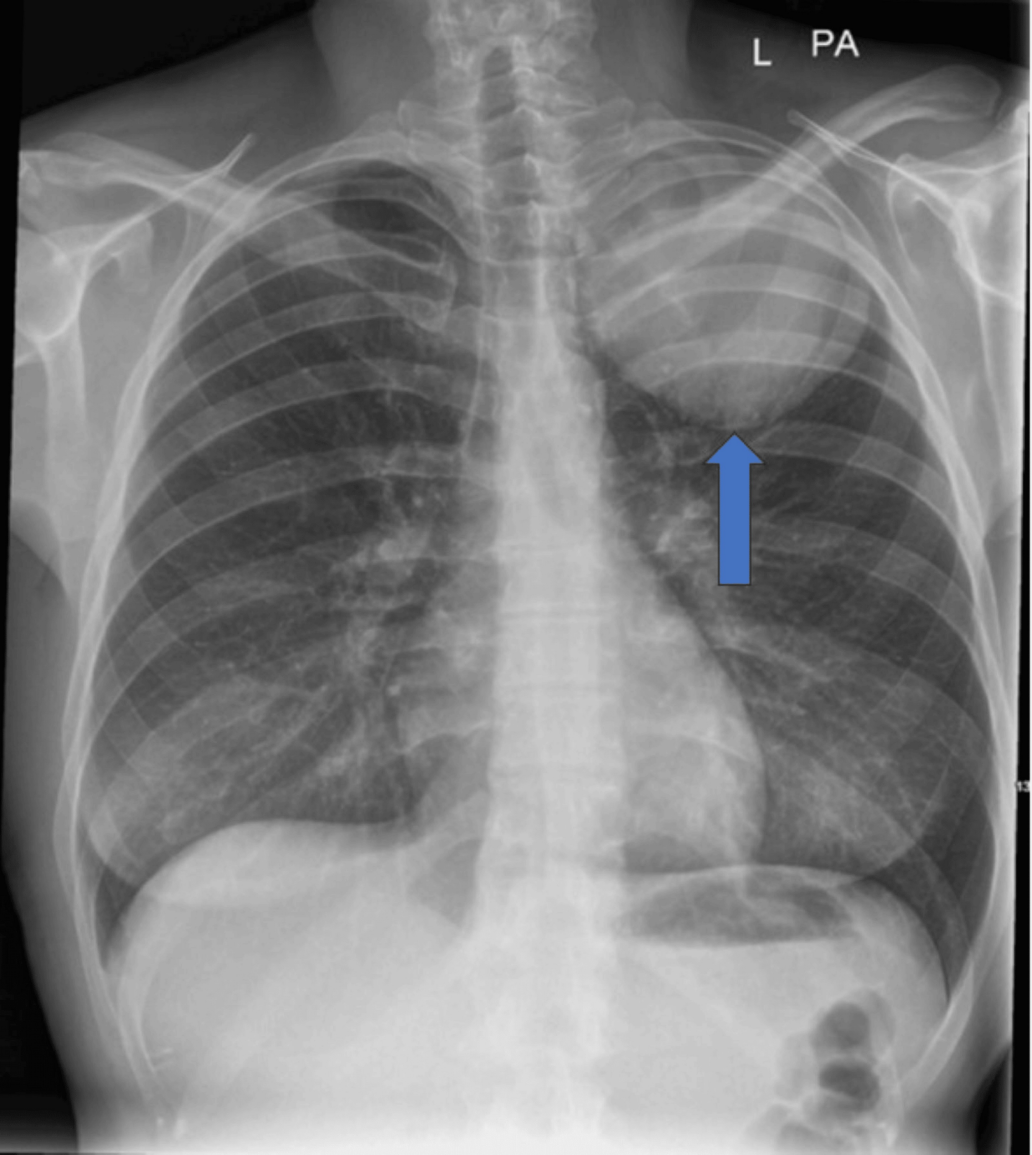
Chest X-ray showing a 10 cm rounded homogenous opacity at the left apex

**Figure 2:**
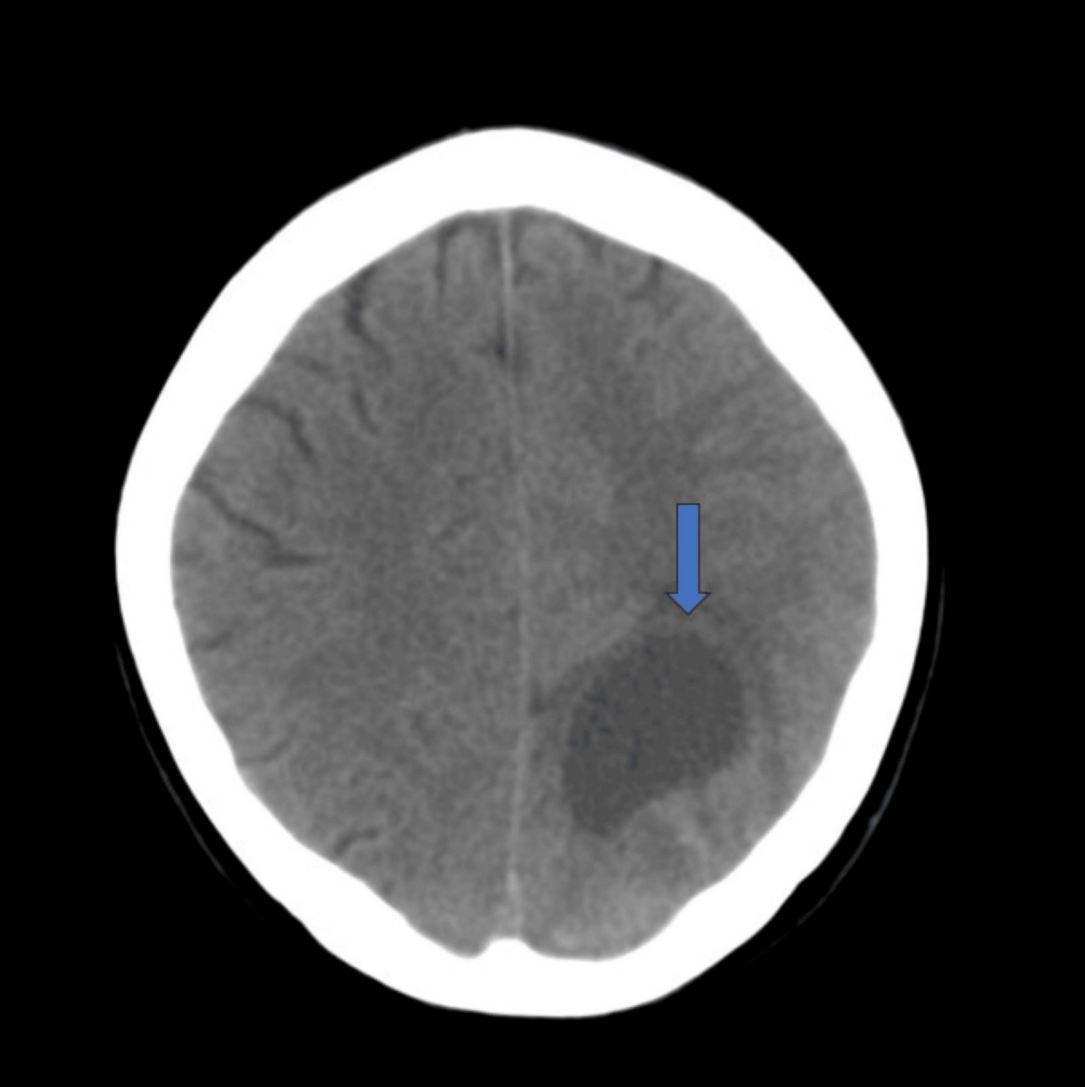
CT of the head showing a 4.5 cm rim-enhancing cystic lesion in the left occipital lobe and extensive surrounding edema and mass effect along with a left middle cerebral artery (MCA) bifurcation aneurysm

Physical examination revealed faint crackles at the left lung apex, normal heart sounds, and mild right upper quadrant abdominal tenderness. Neurological examination showed left homonymous hemianopia with no lateralizing sign. A chest X-ray revealed a 10 cm rounded opacity in the left upper lung zone (Figure 1). A CT scan of the head showed a 4.5 cm rim-enhancing lesion in the left occipital lobe, along with a left middle cerebral artery (MCA) aneurysm (Figure 2). Her serum beta-human chorionic gonadotropin (β-HCG) level was significantly elevated at 5,642 mIU/mL, although abdominal and pelvic ultrasound showed no evidence of intrauterine or extrauterine pregnancy or masses. HIV and TB tests were both negative.

**Figure 3:**
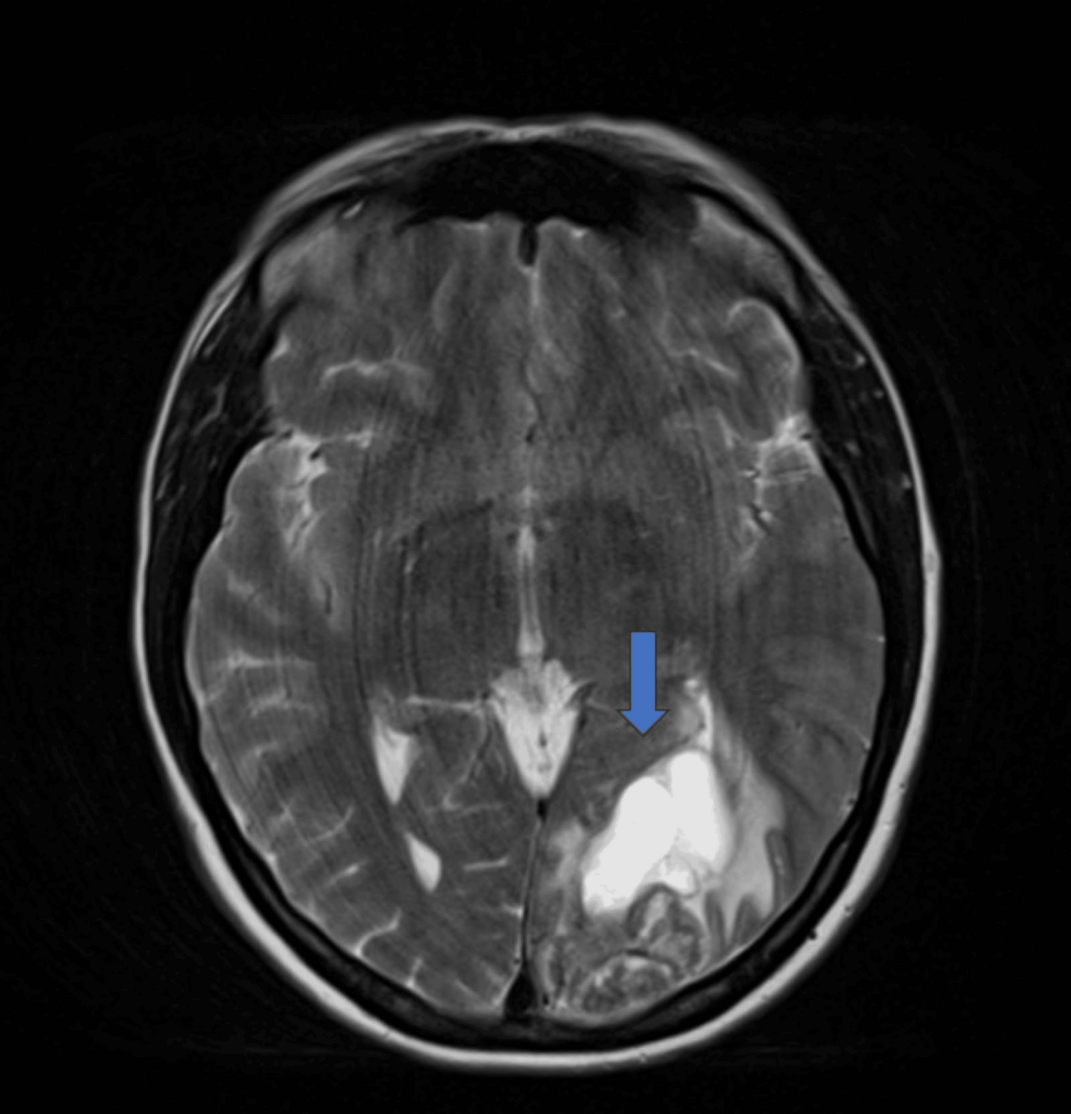
Magnetic resonance imaging (MRI) showing a solitary/cystic lesion in the left parieto-occipital lobe with peripheral enhancement and hemorrhage within

Initially, she was started on ceftriaxone, vancomycin, and metronidazole for a possible cerebral abscess. Respiratory consultation recommended a CT thorax, abdomen, and pelvis (CT TAP) and magnetic resonance imaging (MRI) of the brain. Brain MRI with contrast revealed an isolated solid/cystic lesion in the left parieto-occipital lobe with peripheral enhancement and hemorrhage, initially suggestive of glioblastoma (Figure 3). CT TAP showed a large apical mass with underlying emphysema and mild mural thickening of the gastric pylorus but no definitive primary lesion or metastatic disease and, of significance, no ovarian/adnexal mass.

The neurology team recommended that she consult neurosurgery, which advised starting dexamethasone and Keppra and transferring her for further care. The patient underwent a left occipital craniotomy with resection of the lesion, with no intraoperative or postoperative complications. Histopathology confirmed metastatic choriocarcinoma. She was subsequently started on induction chemotherapy with etoposide and cisplatin (EP) for three cycles. STR genotyping confirmed the diagnosis of NGCO, and she was transitioned to bleomycin, etoposide, and cisplatin (BEP) chemotherapy. Pulmonary function tests (PFTs) showed a diffusing capacity of the lungs for carbon monoxide (DLCO) of 70% before starting BEP.

After two cycles of BEP, the patient β-HCG levels decreased from 7,000 mIU/mL to 200 mIU/ml. However, subsequent serial β-HCG measurements demonstrated a plateau at 200 mIU/mL within the following weeks. A restaging CT of the brain and CT TAP revealed persistent disease at the site of the resected left occipital brain metastasis, along with a persistent large mass in the upper lobe of the lung. As a result, her chemotherapy regimen was switched to second-line paclitaxel, cisplatin, and ifosfamide (TIP), and she was scheduled for stem cell transplantation due to an incomplete response to TIP chemotherapy. The rationale for this decision to start induction chemotherapy using the EP weekly regimen was based on the National Cancer Control Programme (NCCP) protocols for high-risk gestational trophoblastic disease (GTD) with brain and large pulmonary metastasis.

## Discussion

Pure NGCO is exceedingly rare and arises from gonadal and extragonadal germ cells without any association with pregnancy. While it is rarely found in postmenopausal women, it predominantly affects adolescents and young women [7]. In contrast, primary NGCO often occurs in conjunction with other germ cell tumors, such as teratomas, endodermal sinus tumors, embryonal carcinomas, and dysgerminomas [8].

Non-gestational ovarian choriocarcinoma shares histological similarities with primary GCO, typically linked to pregnancy. However, these two conditions can be differentiated through DNA analysis, where the detection of paternal DNA within the tumor points to a gestational (placental) origin [9].

In our case, the patient presented with advanced stage IV non-gestational extragonadal choriocarcinoma, a malignancy that often exhibits nonspecific symptoms, making misdiagnosis more likely. Imaging confirmed lung and brain involvement, but the primary origin was uncertain. At the same time, β-HCG levels were markedly elevated at 5,642 mIU/mL despite the absence of pregnancy or detectable pelvic/ovarian masses on ultrasound and CT scans. NGCO frequently metastasizes to distant sites. In this case, the lungs and brain were invaded, as demonstrated in previous cases [10,11].

A review of NGCO cases indicates that most present with gynecological symptoms or pelvic masses. In this instance, the absence of such findings posed a diagnostic challenge. Therefore, β-HCG levels and DNA sequencing play a critical role in the accurate diagnosis and effective monitoring of treatment response [12,13].

The BEP chemotherapy regimen remains the standard treatment for germ cell tumors; however, surgical intervention can be advantageous, as demonstrated by our case involving craniotomy and resection. Most reported cases have adhered to this chemotherapy protocol [14]. Studies demonstrated that in cases where β-HCG levels do not respond to first-line BEP chemotherapy, second-line TIP chemotherapy can effectively reduce β-HCG levels and improve overall survival outcomes [15].

## Conclusions

NGCO is a rare and challenging condition to diagnose, as its clinical presentation can often be misleading. In our case, the patient presented with both brain and lung masses, highlighting the critical role of serial β-HCG measurements in aiding the diagnosis of GCO. Furthermore, DNA sequencing plays a pivotal role in differentiating between gestational and NGCO, ensuring accurate treatment. Given the aggressive nature and rapid progression of this malignancy, early diagnosis, along with a multidisciplinary approach, is essential to improving patient outcomes and overall prognosis.
